# It Is Not Carved in Stone—The Need for a Genetic Reevaluation of Variants in Pediatric Cardiomyopathies

**DOI:** 10.3390/jcdd9020041

**Published:** 2022-01-25

**Authors:** Dominik Sebastian Westphal, Kathrin Pollmann, Christoph Marschall, Annette Wacker-Gussmann, Renate Oberhoffer-Fritz, Karl-Ludwig Laugwitz, Peter Ewert, Cordula Maria Wolf

**Affiliations:** 1 Department of Internal Medicine I, Klinikum rechts der Isar, School of Medicine & Health, Technical University Munich, 81675 Munich, Germany; sekretariat1M-Laugwitz@mri.tum.de; 2 Institute of Human Genetics, Klinikum rechts der Isar, School of Medicine & Health, Technical University Munich, 81675 Munich, Germany; 3 DZHK (German Centre for Cardiovascular Research), Partner Site Munich Heart Alliance, 80802 Munich, Germany; ewert@dhm.mhn.de (P.E.); wolf@dhm.mhn.de (C.M.W.); 4 Department of Congenital Heart Defects and Pediatric Cardiology, German Heart Center Munich, School of Medicine & Health, Technical University of Munich, 80636 Munich, Germany; pollmann.k@web.de (K.P.); annette.wacker-gussmann@tum.de (A.W.-G.); renate.oberhoffer@tum.de (R.O.-F.); 5 Department of Molecular Genetics, MVZ Martinsried GmbH, 82152 Martinsried, Germany; Christoph.Marschall@medizinische-genetik.de; 6 Institute of Preventive Pediatrics, Department of Sport and Health Sciences, Technical University Munich, 80992 Munich, Germany

**Keywords:** pediatric, childhood, cardiomyopathy, genetic, variant, molecular genetic diagnosis

## Abstract

(1) Background: In cardiomyopathies, identification of genetic variants is important for the correct diagnosis and impacts family cascade screening. A classification system was published by the American College of Medical Genetics and Genomics (ACMG) in 2015 to standardize variants’ classification. The aim of the study was to determine the rate of reclassification of previously identified variants in patients with childhood-onset cardiomyopathies. (2) Methods: Medical records of patients and their relatives were screened for clinical and genetic information at the Department of Congenital Heart Defects and Pediatric Cardiology, German Heart Center Munich. Patients without an identified genetic variant were excluded from further analyses. Previously reported variants were reevaluated by the ACMG criteria in November 2021. (3) Results: Data from 167 patients or relatives of patients with childhood-onset cardiomyopathy from 137 families were analyzed. In total, 45 different genetic variants were identified in 71 individuals. Classification changed in 29% (13/45) with the greatest shift in “variants of unknown significance” to “(likely) benign” (9/13). (4) Conclusions: In patients with childhood-onset cardiomyopathies, nearly a third of reported genetic variants change mostly to more benign classes upon reclassification. Given the impact on patient management and cascade screening, this finding underlines the importance of continuous genetic counseling and variant.

## 1. Introduction

Cardiomyopathies can be classified according to their origin in primary or secondary cardiomyopathies, as well as based on their morphological presentation [1]. Prominent examples for the latter are hypertrophic (HCM), dilated (DCM), and arrhythmogenic cardiomyopathy (ACM) [2]. Cardiomyopathies can occur at different ages, but especially, cardiomyopathy with early onset in childhood can be severe and is often caused by de novo genetic variants [3]. The overall genetic diagnostic yield in cardiomyopathies depends on the morphology with up to 60% in HCM and only 10–25% in the case of isolated DCM [4]. The genetic diagnosis, however, is important in regard to the recurrence risk, but it also has consequences for the clinical treatment and prognosis [3] and impacts cascade screening of family members. Due to the growing number of disease genes, broad genetic testing by next-generation sequencing became the favored tool in diagnostics of cardiomyopathies in adults [5] and pediatric patients [3].

The possibility of broad sequencing, however, comes along with an increasing number of detected variants. In order to rate these variants in the context of their pathogenicity, the “Standards and Guidelines for the Interpretation of Sequence Variants” were created and published by the American College of Medical Genetics and Genomics (ACMG) in 2015 [6]. Based on this rating, the genetic diagnosis can be established in the index patient, and further relatives can be offered predictive testing. Before 2015, there was no commonly shared rating system. Additionally, even if classified according to the ACMG criteria, a variant’s rating can change due to new published scientific knowledge. Consequently, regular variant reevaluation can lead to a shift from a disease-causing variant to a variant of unknown significance (VUS) in a significant proportion of cases, as our group has previously shown for inherited long QT syndrome [7]. The previously reported findings of reclassification in 10% of variants causing HCM [8] and of a noticeably decreased number of uncertain findings (i.e., VUS) in patients with ACM by variants’ reassessment [9] also underlines the importance of periodic reassessment of pathogenicity of variants involved in cardiomyopathies. In pediatric DCM, the largest shift of variant reclassification was observed from inconclusive findings to negative results [10]. These findings come together with benefits for relatives such as less frequently required cardiological follow-ups [11].

In accordance with these previous studies, we aimed to evaluate the rate of reclassified variants in our cohort of mixed pediatric cardiomyopathies.

## 2. Materials and Methods

The study was performed according to the declaration of Helsinki. All patients or their guardians, respectively, gave written consent for the anonymous publication of their data. The study was approved by the institution’s ethical committee (Approval Number 243/17S from 16 October 2017).

### 2.1. Clinical Study

Medical records of patients diagnosed with pediatric-onset cardiomyopathy and their relatives were retrospectively screened for clinical and molecular genetic information at the Department of Congenital Heart Defects and Pediatric Cardiology, German Heart Center Munich. Patients without an identified genetic variant were excluded from further analyses.

### 2.2. Variant Interpretation

Variants were reevaluated according to the ACMG criteria (2015) [6] in November 2021. The variant interpretation was conducted as described before [7] and, at the earliest, two years after the initial report. For the interpretation of loss-of-function (LoF) variants, additional recommendations were used [12]. Information regarding previous interpretations was received from the ClinVar database (https://www.ncbi.nlm.nih.gov/clinvar/, last access on 24 November 2021) and the Human Gene Mutation Database (HGMD^®^) Professional. A PubMed-based (https://www.ncbi.nlm.nih.gov/pubmed, last access on 24 November 2021) review of the literature was conducted if required. The gnomAD dataset was used for variant frequencies in controls (https://gnomad.broadinstitute.org/, last access on 24 November 2021). The gnomAD gene constraints were used to estimate the impact of LoF or missense variants on gene function. For applying the criterion “absent from healthy controls” (PM2), the previously recommended allele frequency of <4 × 10^−5^ was used as cut-off [13]. The following in silico prediction scores were used, additionally: CADD (https://cadd.gs.washington.edu/snv, last access on 24 November 2021), SIFT (https://sift.bii.a-star.edu.sg/, last access on 24 November 2021), and PolyPhen-2 (http://genetics.bwh.harvard.edu/pph2/, last access on 24 November 2021).

### 2.3. Statistics

Statistical analysis was performed with the SPSS software version 28.0.0 (SPSS Inc.; IBM Company, Chicago, IL, USA). Based on the small study population normal distribution of variables could not be assumed and only nonparametric tests were used. Continuous variables are expressed as median (minimum–maximum), and comparison between groups was performed by Mann–Whitney U test when analyzed between the groups. Categorical variables were given as the total number of cases and as percentages of group totals, comparisons were made by Pearson’s chi-squared test, where appropriate. A *p*-value of less than 0.05 (two sided) was considered statistically significant.

## 3. Results

Data from a total of 167 patients or relatives of patients with childhood-onset cardiomyopathy from 137 families were analyzed. The patients were examined in our department in the years between 1979 and 2021. Most of the patients were affected by HCM (75/167). Other cardiomyopathies comprised DCM (12/167), left ventricular noncompaction cardiomyopathy (LVNC, 12/167), ACM (3/167), and restrictive cardiomyopathy (2/167). HCM associated with Noonan syndrome or Noonan syndrome with multiple lentigines (NSML) was diagnosed in 26/167 patients. One patient was affected by Morbus Fabry. In addition, 36 patients were screened as family members and did not express any clinical phenotype. Family history was positive in 102 patients, negative in 57 patients, and not available in 8 patients. A genetic examination was performed in 75.5% (126/167), at a median age of 16.4 years (range 0–67.9 years). The median age of the pediatric patients at their first genetic test was 7.1 years (range 0–17.8 years). The genetic diagnostics were performed in the years between 2009 and 2019. Type of molecular genetic testing included panel diagnostic in 45, exome sequencing in 13, and targeted diagnostics in 42 patients. The method of genetic testing was not known in 26 cases. A total of 45 different genetic variants were identified in 71 individuals (positive genetic findings are summarized in Table S1). Exact nucleotide or protein exchange was not available in 21 additional patients. Therefore, these patients were excluded from the reanalysis. In 26 index cases, there was no variant detected by genetic testing. Eight additional individuals were unaffected relatives that were tested for a known familial pathogenic variant, but the variant could not be detected. All genetic findings are summarized in Table 1.

### 3.1. A Total of 29% of Variants Were Significantly Reclassified

**Figure 1:**
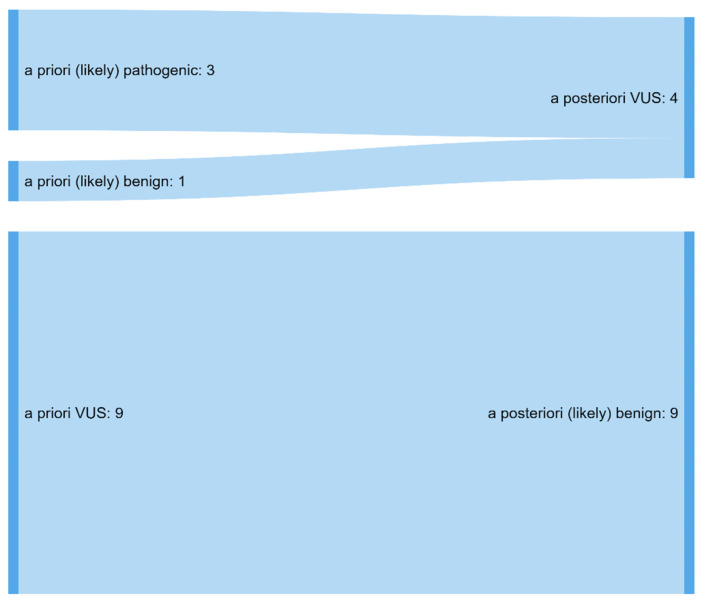
Classification changed after reassessment in a total of 13 variants. Most classifications shifted from VUS to likely benign. VUS: variant of unknown significance.

Most of the 45 different genetic variants previously identified in a total of 71 individuals were missense variants (35/45), followed by splice (4/35) and frameshift variants (3/35), as well as one stop variant, synonymous variant, and in-frame deletion (Table S1). Reevaluation of these variants was performed at a median (range) of 4.4 years (2–15.4 years) after the initial report. On reevaluation of the previously reported variants, classification changed significantly (as described in the Methods Section) in 28.9% (13/45). In total, 13 different variants affected 7 genes and were detected in 12 different individuals, including 3 members of one family (patient IDs 125, 145, 172) and two patients that carried the same variant (patient IDs 60, 146). Most of the classification changed from VUS to (likely) benign (9/13, Figure 1). All reclassified were missense variants and mostly reported in 2015 or later (11/13) (overview of all reclassified variants in Table 3). There was no difference between the length of time from the first report to reclassification in the cases in which variants were reclassified, compared with those in which classification remained identical (median (range) time between the first report and reclassification 5.8 (3.1–7.2) years and 4.8 (2.6–12.6) years, respectively, *p* = 0.806, Mann–Whitney–Wilcoxon test).

### 3.2. The Number of Detected Variants in One Patient Correlates with the Chance of Reclassification

Out of the 10 cases with a reclassified variant (patients 125, 145, and 172 from the same family counted as one case), one further variant that might explain the phenotype was identified in 4 cases (40%). Family history had no significant influence on the reclassification of variants (*p* = 0.858, Pearson’s chi-squared test). Most of the cases were affected by HCM (7/10), which proposed the largest subgroup in our cohort (75/167). Although patients with Noonan syndrome or NSML represented the second-largest clinical subgroup (26/167), apart from patients with no clinical phenotype (36/167), no variants were reclassified in both subgroups. Two cases with reclassified variants were affected by DCM and one by LVNC.

## 4. Discussion

Variant interpretation is especially challenging in patients with cardiomyopathies. It is commonly known that cardiomyopathies have variable expressivity and disease penetrance [14,15]. These findings might be partly explained by the cumulative effect of common variants, measured by polygenic risk scores [16,17]. These polymorphisms, however, escape the ACMG classification system, which was created for monogenic disorders. As a result, disease-causing variants can be found in healthy controls, such as the gnomAD dataset, which might lead to exclusion of the moderate criterion PM2 (absent from healthy controls) or even application of the strong benign criterion BS1 (allele frequency is greater than expected for the disorder). There are, for example, heterozygous carriers (patient IDs 103, 104) of the variant c.3613G > A, *p*. (Glu1205Lys) in *MYH7* in the gnomAD database (variant ID: 14-23889167-C-T). PM2 was applied in our study as recommended before [13] since subclinical cardiomyopathy cannot be excluded in this single individual. Its application, however, is disputable and, moreover, decisive for the variant’s classification as “likely pathogenic”. Another problem arises from the fact that control data depends on ethnic background. Rare variants in a distinct population can be common in another group. This can lead to misinterpretation in the context of pathologization of benign variants, as it was shown for HCM patients with African ancestry [18].

In order to address these difficulties, different approaches were developed. There were, for example, specific guidelines elaborated for variant interpretation in DCM, such as the stand-alone benign criterion “BP1_Stand_Alone” in case of a missense variant in the *TTN* gene [19]. This criterion reflects the problem with *TTN*: The encoded protein Titin is a large structural protein with different isoforms [20] that tolerates numerous numbers of missense variants. There are even more observed than expected missense variants in the gnomAD database (z-score: −1.1). Even loss-of-function variants are tolerated to a certain degree (gnomAD LOEUF = 0.35). Therefore, it was proposed that PVS1 should only be applied as a strong criterion in case of a null variant in the A band of *TTN* [19], where truncation variants cluster in DCM patients [20]. In HCM, an algorithm was developed based on the clustering of variants in specific gene regions [13], as well as the clinical “Mayo Score” for improving the pre-test probability in genetic testing of affected patients [21]. Although these are promising approaches, the problem remains that these tools are often not as commonly used as the well-established ACMG criteria. Time will tell which of these systems will be adopted by laboratories in the future.

In our cohort with pediatric-onset cardiomyopathy, initial variant classification changed in a considerable proportion of 28.9% (13/45) after reassessment. This finding is remarkable, considering that most cases were affected by HCM and the variants’ classification changed in 10% of patients with HCM in a previous study [8]. One explanation for this discrepancy might be that we analyzed a mixed cohort of different cardiomyopathies. Moreover, 77.8% of the initially reported variants were missense variants, which have an intrinsic uncertainty regarding their interpreted pathogenicity. However, the greatest shift in our cohort was from VUS to likely benign variants, which is in concordance with a previous study of patients with DCM in which also about 30% of VUS were reclassified as (likely) benign [10]. The patients’ family history had no statistical effect on the variants’ reclassification, although this might not be reliable due to the relatively small number of reclassified variants. Interestingly, the length between first diagnosis and last clinical follow-up was significantly longer in patients with reclassification than in patients without a change in the classification. This might reflect that the growing chance of reclassification is dependent on the passed time since the last consultation (limited by the small group of patients with reclassified variants). The time between the first genetic report and reclassification, however, was not significantly longer in both groups.

The performed reclassification is not only important for clinicians in order to establish the correct diagnosis. It also has a direct impact on the patients and their decisions and lifestyle. Additionally, molecular genetic findings have direct consequences for predictive testing of additional relatives and decisions for prenatal testing or even pre-implantation genetic diagnosis. Therefore, the possibility of variant reclassification should always be carefully discussed in genetic counseling, in the case of VUS, as well as when reporting (likely) pathogenic variants [22]. In cases with reclassified variants, such as the ones reported in this study, the next step is informing the patients and their relatives about the findings through genetic counseling. In case of a shift from a likely pathogenic variant to a more benign class, repetition of genetic diagnostics is offered to identify another, disease-causing variant that might have been missed. For relatives who are not under cardiological evaluation because of previous exclusion of such a variant, cardiological follow-ups are recommended, again, dependent on their echocardiographic status. Genetic counseling and testing are performed according to previously published recommendations [23]. Cardiological follow-ups and treatment are continued according to the different clinical guidelines or recommendations, respectively [1,24,25,26,27]. The reclassification of a variant will most likely have no direct consequence on the clinical treatment of our index patients since the recommendations are primarily based on clinical findings. However, general consequences of falsely classified variants can be a different risk assessment due to variants in pro-arrhythmic disease genes or psychological stress for relatives that were tested predictively tested for this variant.

## 5. Conclusions

While next-generation sequencing makes wide genetic diagnostics available, variant interpretation remains challenging. Especially in diseases with low penetrance and broad expressivity, such as cardiomyopathies, the possibility of variant reclassification should always be taken into consideration when counseling patients and their relatives.

## Figures and Tables

**Table 1:** Genetic findings.

N = 167	HCM n = 75	DCM n = 12	ACM n = 3	LVNC n = 12	RCM n = 2	Noonan Syndrome/NSML n = 26	Morbus Fabry n = 1	No Clinical Phenotype n = 36
Male n = 101	48	7	2	8	1	16	0	19
Positive family history n = 102	44	7	2	6	2	4	1	36
Patients without genetic testing n = 41	15	4	1	3	0	3	0	15
Patients with positive genetic findings n = 71	30	6	2	7	0	15	0	11
Initial classification of variants N = 45 *								
Likely benign (class II) n = 2	1	0	0	1	0	0	0	0
VUS (class III) n = 8	2	3	0	2	0	0	0	1
Likely pathogenic (class IV) n = 14	9	2	0	1	0	1	0	1
Pathogenic (class V) n = 21	9	0	1	1	0	8	0	2

* Number of different variants, ACM: arrhythmogenic cardiomyopathy, DCM: dilated cardiomyopathy, HCM: hypertrophic cardiomyopathy, LVNC: left ventricular noncompaction cardiomyopathy, NSML: Noonan syndrome with multiple lentigines, RCM: restrictive cardiomyopathy. Of the 71 patients with an identified variant, the median age at first diagnosis was 4.7 years (range 0–50.3 years). Median New York Heart Association (NYHA) classification was 1 (range 1–2.5) at last clinical follow-up, and patients presented with mainly preserved ejection fraction (median 70 percent, range 25–94 percent). The length between first diagnosis and last clinical follow-up was significantly longer in patients with reclassified variants (median 15.9 years, range 7.4–26 years) than in patients without change in the classification (median 6.1 years, range 0–33.5 years, *p* = 0.047). Clinical findings are summarized in Table 2.

**Table 2:** Clinical findings of patients with identified molecular genetic diagnosis.

Variable	All Patients (n = 71)	No Reclassification (n = 59)	Reclassification (n = 7) ^†^	*p*-Value
Age at first diagnosis (years) *	4.7 [0–50.3]	5.5 [0–50.3]	0 [0–12]	0.219
Length between first diagnosis and last clinical follow-up (years) *	7.7 [0–33.5]	6.1 [80–33.5]	15.9 [7.4–26]	0.047
NYHA/Ross *	1 [1–2.5]	1 [1–2.5]	1.5 [1–1.5]	0.596
Ejection fraction (%) *	70 [25–94]	70 [25–94]	69 [61–81]	0.863
Number of cardiac medications *	0 [0–5]	0 [0–5]	0.5 [0–2]	0.805
Death	No deaths			
Hospital stay **^,†^	26/58 (44.8%)	24/52 (46.2%)	2/6 (33.3%)	0.681
ICD implantation **^,†^				
Primary prophylactic	7/68 (10.3%)	6/61 (9.8%)	1/7 (14.3%)	0.550
Secondary prophylactic	None			
Appropriate ICD discharge **^,†^	5/7 (7.1%)	5/6 (83.3%)	1/1 (100%)	0.286
Cardiac surgery **^,†^	13/68 (19.1%)	11/61 (18%)	2/7 (28.6%)	0.611

* Median (range): non-parametric Mann–Whitney–Wilcoxon test, ** n/N (%): Fisher’s Exact test, ^†^ complete clinical data was not available in a subset of patients; ICD: implantable cardioverter defibrillator.

**Table 3:** Overview of reclassified variants.

Patient ID	Phenotype	Variant	Original Classification	Reclassification	Applied ACMG Criteria [6]	Year of Initial Report	Further Variants
31	HCM	*TPM1* (NM_001018005.1): c.287A > G, p.(Glu96Gly)	LP	VUS	PM1, PP3, PP5	2014	-
49	LVNC	*TTN* (NM_001267550.2): c.97612C > T, p.(Arg32538Cys)	VUS	LB	BS1, BP6	2019	VUS in *TTN*, B variant in *LMNA*
49	LVNC	*LMNA* (NM_170707.3): c.1930C > T, p.(Arg644Cys)	VUS	B	PS3, BS1, BS4, BP6	2019	VUS and LB variant in *TTN*
60	HCM	*MYBPC3* (NM_000256.3): c.1468G > A, p.(Gly490Arg) **	LP	VUS	PP3, PP5, BS1	unknown	-
61	HCM	*MYBPC3* (NM_000256.3): c.405A > G, p.(Lys135 = )	VUS	LB	BS3, BP6, PM2	2018	-
65	DCM	*TTN* (NM_001267550.2): c.94851T > A, p.(Asp31617Glu)	VUS	LB	BS1, BP4, BP6	2015	LB and B variants in *TTN*
65	DCM	*TTN* (NM_001267550.2): c.14870C > G, p.(Thr4957Ser)	VUS	LB	BS1, BP6	2015	LB and B variants in *TTN*
65	DCM	*DSG2* (NM_001943.3): c.1174G > A, p.(Val392Ile)	VUS	B	BS1, BS3, BP4, BP6	2015	Two LB variants in *TTN*
121	HCM	*DSP* (NM_004415.4): c.7655T > C, p.(Leu2552Pro)	LB	VUS	PM2, PP3	2018	-
124	HCM	*MYBPC3* (NM_000256.3): c.2992C > G, p.(Gln998Glu)	VUS	LB	PP2, BS1, BP6	unknown	-
125, 145, 172 *	DCM	*MYOZ2* (NM_016599.5): c.713G > T, p.(Gly238Val)	LP	VUS	PM2, PP3	2015	P variant in *DSP*
146	HCM	*MYBPC3* (NM_000256.3): c.1468G > A, p.(Gly490Arg) **	LP	VUS	PP3, PP5, BS1	2016	-
168	HCM	*TTN* (NM_001267550.2): c.19738C > T, p.(Pro6580Ser)	VUS	LB	BS1, BP6	2017	VUS in *MYH6*, LB variant in *TTN*
168	HCM	*TTN* (NM_001267550.2): c.18961A > G, p.(Ile6321Val)	VUS	LB	BS1, BP4, BP6	2017	VUS in *MYH6*, LB variant in *TTN*

* Members of the same family, ** same variant, B: benign, LB: likely benign, LP: likely pathogenic, VUS: variant of unknown significance.

## Data Availability

The data that support the findings of this study are available from the corresponding author upon reasonable request.

## Supplementary Materials

The following supporting information can be downloaded at: https://www.mdpi.com/article/10.3390/jcdd9020041/s1, Table S1: List of classified variants.

## References

[1] Ommen S.R., Mital S., Burke M.A., Day S.M., Deswal A., Elliott P., Evanovich L.L., Hung J., Joglar J.A., Kantor P. (2020). 2020 AHA/ACC Guideline for the Diagnosis and Treatment of Patients With Hypertrophic Cardiomyopathy: A Report of the American College of Cardiology/American Heart Association Joint Committee on Clinical Practice Guidelines. Circulation.

[2] McKenna W.J., Maron B.J., Thiene G. (2017). Classification, Epidemiology, and Global Burden of Cardiomyopathies. Circ. Res..

[3] Vasilescu C., Ojala T.H., Brilhante V., Ojanen S., Hinterding H.M., Palin E., Alastalo T.P., Koskenvuo J., Hiippala A., Jokinen E. (2018). Genetic Basis of Severe Childhood-Onset Cardiomyopathies. J. Am. Coll. Cardiol..

[4] Hershberger R.E., Givertz M.M., Ho C.Y., Judge D.P., Kantor P.F., McBride K.L., Morales A., Taylor M.R.G., Vatta M., Ware S.M. (2018). Genetic evaluation of cardiomyopathy: A clinical practice resource of the American College of Medical Genetics and Genomics (ACMG). Genet. Med..

[5] Akinrinade O., Ollila L., Vattulainen S., Tallila J., Gentile M., Salmenpera P., Koillinen H., Kaartinen M., Nieminen M.S., Myllykangas S. (2015). Genetics and genotype-phenotype correlations in Finnish patients with dilated cardiomyopathy. Eur. Heart J..

[6] Richards S., Aziz N., Bale S., Bick D., Das S., Gastier-Foster J., Grody W.W., Hegde M., Lyon E., Spector E. (2015). Standards and guidelines for the interpretation of sequence variants: A joint consensus recommendation of the American College of Medical Genetics and Genomics and the Association for Molecular Pathology. Genet. Med..

[7] Westphal D.S., Burkard T., Moscu-Gregor A., Gebauer R., Hessling G., Wolf C.M. (2020). Reclassification of genetic variants in children with long QT syndrome. Mol. Genet. Genom. Med..

[8] Das K.J., Ingles J., Bagnall R.D., Semsarian C. (2014). Determining pathogenicity of genetic variants in hypertrophic cardiomyopathy: Importance of periodic reassessment. Genet. Med..

[9] Vallverdu-Prats M., Alcalde M., Sarquella-Brugada G., Cesar S., Arbelo E., Fernandez-Falgueras A., Coll M., Perez-Serra A., Puigmule M., Iglesias A. (2021). Rare Variants Associated with Arrhythmogenic Cardiomyopathy: Reclassification Five Years Later. J. Pers. Med..

[10] Quiat D., Witkowski L., Zouk H., Daly K.P., Roberts A.E. (2020). Retrospective Analysis of Clinical Genetic Testing in Pediatric Primary Dilated Cardiomyopathy: Testing Outcomes and the Effects of Variant Reclassification. J. Am. Heart Assoc..

[11] Towbin J.A. (2020). Pediatric Primary Dilated Cardiomyopathy Gene Testing and Variant Reclassification: Does It Matter?. J. Am. Heart Assoc..

[12] Abou Tayoun A.N., Pesaran T., DiStefano M.T., Oza A., Rehm H.L., Biesecker L.G., Harrison S.M., ClinGen Sequence Variant Interpretation Working G. (2018). Recommendations for interpreting the loss of function PVS1 ACMG/AMP variant criterion. Hum. Mutat..

[13] Walsh R., Mazzarotto F., Whiffin N., Buchan R., Midwinter W., Wilk A., Li N., Felkin L., Ingold N., Govind R. (2019). Quantitative approaches to variant classification increase the yield and precision of genetic testing in Mendelian diseases: The case of hypertrophic cardiomyopathy. Genome Med..

[14] Sedaghat-Hamedani F., Kayvanpour E., Tugrul O.F., Lai A., Amr A., Haas J., Proctor T., Ehlermann P., Jensen K., Katus H.A. (2018). Clinical outcomes associated with sarcomere mutations in hypertrophic cardiomyopathy: A meta-analysis on 7675 individuals. Clin. Res. Cardiol..

[15] Kayvanpour E., Sedaghat-Hamedani F., Amr A., Lai A., Haas J., Holzer D.B., Frese K.S., Keller A., Jensen K., Katus H.A. (2017). Genotype-phenotype associations in dilated cardiomyopathy: Meta-analysis on more than 8000 individuals. Clin. Res. Cardiol..

[16] Pirruccello J.P., Bick A., Wang M., Chaffin M., Friedman S., Yao J., Guo X., Venkatesh B.A., Taylor K.D., Post W.S. (2020). Analysis of cardiac magnetic resonance imaging in 36,000 individuals yields genetic insights into dilated cardiomyopathy. Nat. Commun..

[17] Harper A.R., Goel A., Grace C., Thomson K.L., Petersen S.E., Xu X., Waring A., Ormondroyd E., Kramer C.M., Ho C.Y. (2021). Common genetic variants and modifiable risk factors underpin hypertrophic cardiomyopathy susceptibility and expressivity. Nat. Genet..

[18] Manrai A.K., Funke B.H., Rehm H.L., Olesen M.S., Maron B.A., Szolovits P., Margulies D.M., Loscalzo J., Kohane I.S. (2016). Genetic Misdiagnoses and the Potential for Health Disparities. N. Engl. J. Med..

[19] Morales A., Kinnamon D.D., Jordan E., Platt J., Vatta M., Dorschner M.O., Starkey C.A., Mead J.O., Ai T., Burke W. (2020). Variant Interpretation for Dilated Cardiomyopathy: Refinement of the American College of Medical Genetics and Genomics/ClinGen Guidelines for the DCM Precision Medicine Study. Circ. Genom. Precis. Med..

[20] Deo R.C. (2016). Alternative Splicing, Internal Promoter, Nonsense-Mediated Decay, or All Three: Explaining the Distribution of Truncation Variants in Titin. Circ. Cardiovasc. Genet..

[21] Bonaventura J., Norambuena P., Tomasov P., Jindrova D., Sediva H., Macek M., Veselka J. (2019). The utility of the Mayo Score for predicting the yield of genetic testing in patients with hypertrophic cardiomyopathy. Arch. Med. Sci..

[22] Wong E.K., Bartels K., Hathaway J., Burns C., Yeates L., Semsarian C., Krahn A.D., Virani A., Ingles J. (2019). Perceptions of genetic variant reclassification in patients with inherited cardiac disease. Eur. J. Hum. Genet..

[23] Ackerman M.J., Priori S.G., Willems S., Berul C., Brugada R., Calkins H., Camm A.J., Ellinor P.T., Gollob M., Hamilton R. (2011). HRS/EHRA expert consensus statement on the state of genetic testing for the channelopathies and cardiomyopathies: This document was developed as a partnership between the Heart Rhythm Society (HRS) and the European Heart Rhythm Association (EHRA). Europace.

[24] Silka M.J., Shah M.J., Silva J.N.A., Balaji S., Beach C.M., Benjamin M.N., Berul C.I., Cannon B., Cecchin F., Writing Committee Members (2021). 2021 PACES Expert Consensus Statement on the Indications and Management of Cardiovascular Implantable Electronic Devices in Pediatric Patients: Executive Summary. Heart Rhythm.

[25] Priori S.G., Blomstrom-Lundqvist C. (2015). 2015 European Society of Cardiology Guidelines for the management of patients with ventricular arrhythmias and the prevention of sudden cardiac death summarized by co-chairs. Eur. Heart J..

[26] Baumgartner H., De Backer J., Babu-Narayan S.V., Budts W., Chessa M., Diller G.P., Lung B., Kluin J., Lang I.M., Meijboom F. (2021). 2020 ESC Guidelines for the management of adult congenital heart disease. Eur. Heart J..

[27] Bosman L.P., Te Riele A. (2022). Arrhythmogenic right ventricular cardiomyopathy: A focused update on diagnosis and risk stratification. Heart.

